# The superior salinity tolerance of bread wheat cultivar Shanrong No. 3 is unlikely to be caused by elevated Ta-sro1 poly-(ADP-ribose) polymerase activity

**DOI:** 10.1093/plcell/koac261

**Published:** 2022-08-18

**Authors:** Sarah Vogt, Karla Feijs, Sebastian Hosch, Raffaella De Masi, Ruth Lintermann, Bernhard Loll, Lennart Wirthmueller

**Affiliations:** Department Biochemistry of Plant Interactions, Leibniz Institute of Plant Biochemistry, Halle (Saale), 06120, Germany; Institute of Biochemistry and Molecular Biology, RWTH Aachen University, Aachen, 52074, Germany; Department Biochemistry of Plant Interactions, Leibniz Institute of Plant Biochemistry, Halle (Saale), 06120, Germany; Department of Plant Biochemistry, Freie Universität Berlin, Dahlem Centre of Plant Sciences, Berlin, 14195, Germany; Department Biochemistry of Plant Interactions, Leibniz Institute of Plant Biochemistry, Halle (Saale), 06120, Germany; Department of Plant Biochemistry, Freie Universität Berlin, Dahlem Centre of Plant Sciences, Berlin, 14195, Germany; Department of Plant Biochemistry, Freie Universität Berlin, Dahlem Centre of Plant Sciences, Berlin, 14195, Germany; Laboratory of Structural Biochemistry, Freie Universität Berlin, Berlin, 14195, Germany; Department Biochemistry of Plant Interactions, Leibniz Institute of Plant Biochemistry, Halle (Saale), 06120, Germany; Department of Plant Biochemistry, Freie Universität Berlin, Dahlem Centre of Plant Sciences, Berlin, 14195, Germany

## Abstract

Structural and biochemical analyses demonstrate that the elevated salinity tolerance of bread wheat cultivar Shanrong No. 3 is unlikely to be caused by elevated Ta-sro1 poly(ADP-ribose) polymerase activity.

Dear Editor,

This letter deals with a 2014 publication in *The Plant Cell* by Liu et al. (2014) suggesting that the superior salinity tolerance of bread wheat (*Triticum aestivum*) cultivar Shanrong No. 3 (SR3) can be attributed to poly (ADPribose) polymerase (PARP) activity of the SIMILAR TO RCD-ONE (SRO) protein encoded by the SR3 allele of *sro1*, Ta-*sro1*. We present a structural analysis of the Ta*-*sro1 PARP domain at 2.1 Å resolution together with *in vitro* and *in vivo* assays of biochemical function, providing strong evidence that Ta-sro1 is unlikely to have PARP activity.

Protein ADP-ribosylation is a post-translational protein modification that is conserved in most eukaryotes and has attracted a growing interest in plant responses to biotic and abiotic stress (Liu et al., 2014; Feng et al., 2015; Kong et al., 2021; Yao et al., 2021). Protein-modifying ADP-ribosyltransferases use nicotinamide adenine dinucleotide (NAD^+^) as a co-substrate to transfer the ADP-ribose moiety of NAD^+^ to an amino acid side chain. In mammals and plants, enzymes of the PARP family act as the main “writer” enzymes of intracellular ADP-ribosylation events although some members of the sirtuin family also catalyze protein ADP-ribosylation (Hottiger, 2015). The human genome codes for 17 PARP proteins. Out of these, 2 proteins lack apparent ADP-ribosyltransferase activity, 11 enzymes are restricted to mono-ADP-ribosylation (i.e. the transfer of a single ADP-ribose onto the target residue), and only 4 family members can catalyze multiple rounds of ADP-ribosylation leading to the formation of poly(ADP-ribose) (PAR) chains on substrate proteins (Hottiger, 2015). The type of enzymatic activity is closely correlated with the conservation of residues in the catalytic PARP domain that are either required for NAD^+^ binding or catalysis. A conserved His hydrogen bonds to the adenine ribose of NAD^+^ and, together with a conserved Tyr that stacks with the nicotinamide ribose ring, forms the basis of the co-substrate binding site (Wahlberg et al., 2012). A catalytic Glu residue acts as a general base to facilitate the nucleophilic attack by the acceptor on the donor ribose (Marsischky et al., 1995). Notably, the Glu residue is dispensable for the initial ADP-ribosylation of an Asp or Glu residue because their side chains can function as intrinsic nucleophiles (Kleine et al., 2008). In contrast, during PAR chain elongation, the Glu is required as a general base to polarize the 2′-hydroxyl group of the acceptor’s adenine ribose. Therefore, the Glu is conserved in all human enzymes with poly-ADP-ribosylation activity, whereas its replacement by other amino acids can limit certain PARP family members to mono-ADP-ribosylation (Kleine et al., 2008; Hottiger, 2015).

Plant genomes typically code for three different PARP proteins with occasional duplication of *PARP* genes leading to expression of different isoforms in several species (Vainonen et al., 2016). In Arabidopsis (*Arabidopsis thaliana*), PARP1 and PARP2 have polymerase activity and this is consistent with complete conservation of the His–Tyr–Glu triad in these enzymes (Feng et al., 2015). In contrast, PARP3 has a Cys–Val–Glu triad, does not bind NAD^+^, and is inactive with respect to catalyzing mono- or poly-ADP-ribosylation reactions (Gu et al., 2019). In addition to canonical PARP enzymes, plants have evolved a second group of proteins that share sequence and structural homology with the catalytic domains of PARPs. Transcriptional co-regulators of the SRO group are characterized by a conserved PARP domain and a C-terminal RST (RCD1 SRO TAF4) domain that binds transcription factors (Jaspers et al., 2010). Although the PARP domain in SRO proteins is strictly conserved, residues of the His–Tyr–Glu triad deviate from the consensus sequence in SRO proteins (Jaspers et al., 2010). In Arabidopsis RADICAL-INDUCED CELL DEATH 1 (RCD1), the founding member of the plant SRO protein family, the catalytic triad is replaced by Leu–His–Asn. RCD1 does not bind NAD^+^ and thus cannot catalyze canonical ADP-ribosyltransferase reactions (Jaspers et al., 2010). Moreover, mutations that alter the cleft of the RCD1 PARP domain that corresponds to the active site of canonical PARPs, do not compromise the biological function of RCD1 in development and oxidative stress tolerance (Wirthmueller et al., 2018). However, a recent report showed that the Arabidopsis SRO2 protein is an NAD^+^-dependent mono-ADP-riboysltransferase (Kong et al., 2021). In SRO2, the His and Tyr residues that coordinate NAD^+^ binding are swapped and this may explain why the protein retains enzymatic activity. Liu et al. (2014) proposed that the bread wheat (*T. aestivum*) Ta-SRO1 protein can catalyze poly-ADP-ribosylation reactions although the His–Tyr–Glu is replaced by Leu–His–His in this protein. Moreover, the study by Liu et al. (2014) identified a Ta-SRO1 proteoform (Ta-sro1) that reportedly exhibits elevated PARP activity and renders wheat cultivar SR3 more tolerant to oxidative and high salinity stress. Ta-*sro1* differs from its parental gene in three single nucleotide polymorphisms, two of which alter the amino acid sequence. Based on protein homology modeling, Liu et al. (2014) predicted that the polymorphism Ala/Thr^343^ maps to the Ta-SRO1 NAD^+^-binding site and could alter its PARP activity. We aimed to understand (1) why Ta-SRO1 retains PARP activity despite nonconservation of the catalytic triad and (2) how the two polymorphisms enhance the enzymatic activity of the Ta-sro1 proteoform that confers elevated salinity tolerance in wheat.

## The presumed active site of Ta-sro1 differs in several key features from canonical PARPs

**Figure 1:**
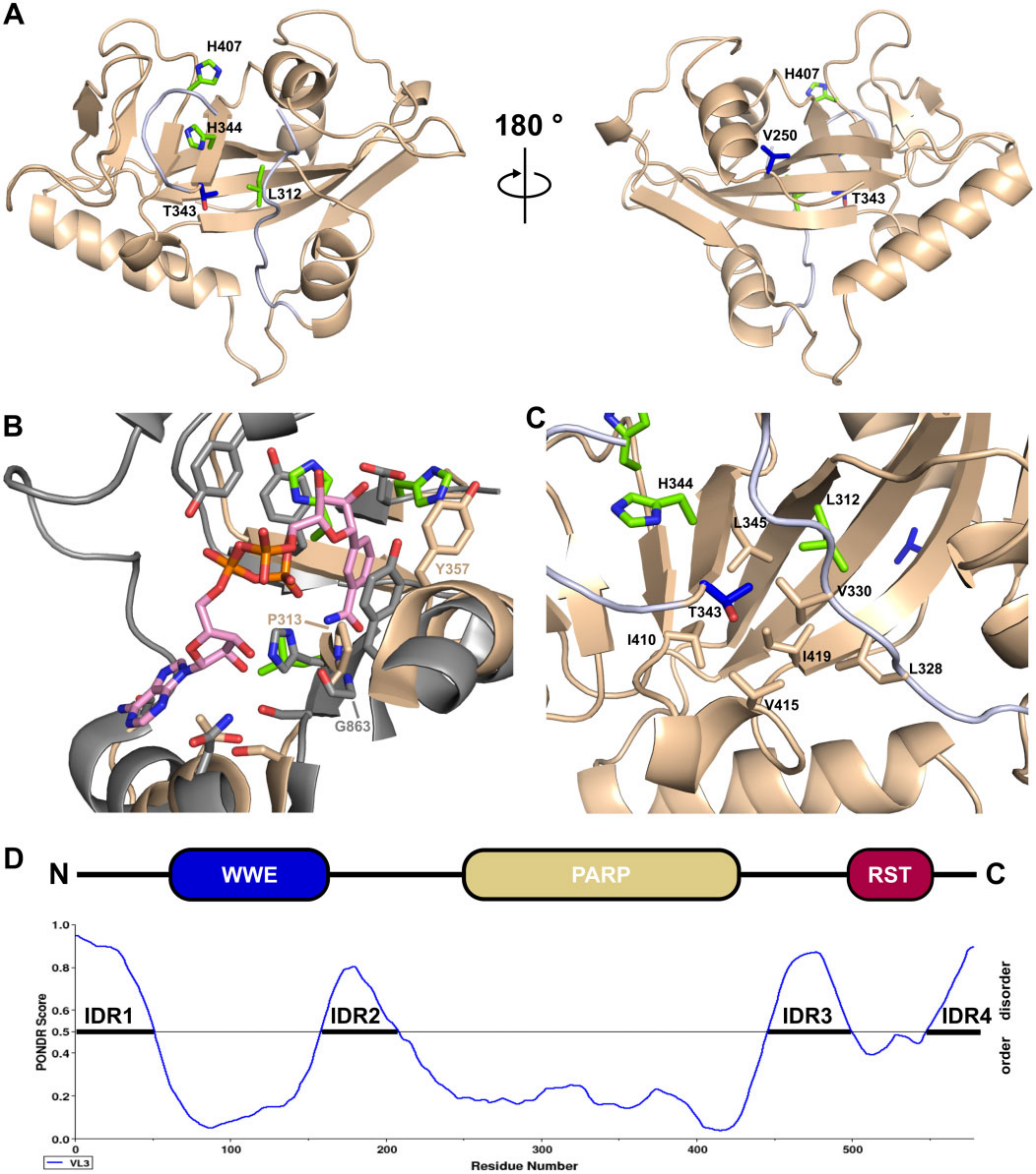
Crystal structure of the Ta-sro1 PARP domain. A, Structure of the Ta-sro1 PARP domain (residues 247–429) in cartoon representation. The postulated catalytic Leu–His–His triad is shown in green. Blue indicates the two polymorphic residues Val^250^ and Thr^343^. The partially disordered D-loop is shown in gray. B, Superposition of the Ta-sro1 PARP domain (beige) and the HsPARP1 PARP domain (gray) co-crystallized in complex with the NAD^+^ analog benzamide adenine dinucleotide (pink) (PDB identifier 6BHV). The position taken by Ta-sro1 residue Pro^313^ is an invariant Gly in catalytically active PARPs. C, The side chain of the polymorphic residue Thr^343^ does not contribute to the postulated NAD^+^-binding site but points towards the hydrophobic core of the PARP domain. D, Ta-sro1 domain architecture and protein disorder profile as predicted by PONDR VL3-BA (Xue et al., 2010). IDRs are labeled IDR1–4. Reflection data and the Ta-sro1 PARP domain structure have been deposited at the Protein Data Bank with identifier 7PLQ. Diffraction images have been deposited at www.proteindiffraction.org under doi:10.18430/M37PLQ. 3D visualizations of protein structures were prepared using PyMol software version 1.7.2 (https://sourceforge.net/projects/pymol/).

To understand the structural basis for the noncanonical PARP activity of Ta-sro1, we produced Selenomethionine-labeled, His_6_-tagged Ta-sro1 PARP domain in *Escherichia coli*, crystallized the protein and solved its structure by single anomalous diffraction phasing (Adams et al., 2010) at 2.1 Å resolution (Protein Data Bank (PDB) identifier 7PLQ; Supplemental Table S1). The domain adopts the typical PARP fold with a β–α–loop–β–α signature at the donor site that binds NAD^+^ (Figure 1A). The donor site loop (D-loop) occludes the proposed active site of Ta-sro1 and is only partially resolved in the structure with little interpretable electron density for amino acids Met^334^–Gly^336^ (Figure 1A). The structure confirms that the His–Tyr–Glu triad is not conserved in Ta-sro1 (Figure 1A). The position of the conserved His residue is taken by Ta-sro1 Leu^312^. Strict conservation of a His residue at this position in active ADP-ribosyltransferases is explained by its function in forming a hydrogen bond to the 2′-OH of the adenine ribose (Ruf et al., 1996; Steffen et al., 2013). Consequently, His in this position is essential for HsPARP1 activity (Marsischky et al., 1995) and a corresponding His^21^ to Leu exchange in the structurally related diphtheria toxin abolishes ADP-ribosylation (Johnson and Nicholls, 1994; Bell and Eisenberg, 1997). The Tyr that stacks with the nicotinamide ribose ring in canonical PARP enzymes is replaced by His^344^ in Ta-sro1. In contrast, a second Tyr that is positioned on the opposite side of the cleft, stacking with the nicotinamide ring, is conserved (Tyr^357^) (Figure 1B). The catalytic Glu that is specifically required for the PAR chain elongating polymerase activity of PARPs is replaced by Ta-sro1 His^407^. Overall, the PARP domain structure reveals that the presumed active site of Ta-sro1 substantially differs in several key residues from canonical PARP domains of plants and mammals (Wahlberg et al., 2012; Vainonen et al., 2016; Gu et al., 2019). Despite the altered identity of amino acids that form the NAD^+^-binding site, Ta-sro1 apparently can still utilize NAD^+^ as a co-substrate (Liu et al., 2014). To understand how Ta-sro1 makes contact to NAD^+^ we superimposed the Ta-sro1 PARP domain onto the structure of the catalytic domain of human PARP1 crystallized in complex with the NAD^+^ analog benzamide adenine dinucleotide (PDB identifier 6BHV). Figure 1B shows the active site of HsPARP1 (gray) with the bound benzamide adenine dinucleotide molecule (pink) in comparison to the presumed Ta-sro1 active site (beige). In addition to the altered identity of several residues that make contact with NAD^+^ (described above), it is apparent that the side chain of Pro^313^ would sterically interfere with NAD^+^ binding if Ta-sro1 would bind the co-substrate in the same orientation as canonical PARPs. The corresponding residue in catalytically active mono- and poly-ADP-ribosyltransferases is an invariant Gly that accommodates the amide group of the nicotinamide moiety and stabilizes it by forming two hydrogen bonds (Wahlberg et al., 2012). Consistently, a corresponding Gly to Trp mutation in HsPARP10 abolishes ADP-ribosyltransferase activity (Yu et al., 2005).

## Side chains of the polymorphic amino acids in Ta-sro1 do not directly contribute to the presumed NAD^+^-binding site

The Ta-sro1 PARP domain structure pinpoints the locations of the two amino acid polymorphisms that distinguish the hypermorphic proteoform Ta-sro1 from Ta-SRO1. Thr^343^ (Ala in Ta-SRO1) is located close to the proposed active site and its side chain is positioned toward the hydrophobic core of the protein formed by residues Leu^345^, Val^330^, Leu^328^, Ile^419^, Val^415^, and Ile^410^ (Figure 1, A and C). In HsPARP1, the corresponding amino acid has been identified as a “gatekeeper” residue that, when mutated, widens the NAD^+^-binding site for access to bulkier NAD^+^ derivatives (Gibson et al., 2016). Based on protein homology modeling, Liu et al. (2014) proposed that the Ala/Thr^343^ side chain interacts with the co-substrate NAD^+^. However, our Ta-sro1 PARP domain crystal structure reveals that the Ta-sro1 Thr^343^ side chain points in the opposite direction and toward a hydrophobic area forming the basis of the postulated NAD^+^-binding site (Figure 1C). Conceivably, exchange of a hydrophobic to a polar amino acid at this site could slightly alter the position of strand β3 that forms one side of the pocket and this might affect the enzymatic activity of Ta-SRO1. The second polymorphic amino acid, Val^250^ (Gly in Ta-SRO1) is positioned on the opposite side of the domain and located close to the N-terminus of the crystallized construct (Figure 1A). In the context of the full-length protein, Val^250^ would form part of the transition from the predicted intrinsically disordered region 2 (IDR2) to the PARP domain (Figure 1D). Understanding the functional relevance of the Gly^250^ to Val exchange, therefore, may require further structural information in the context of the WWE-PARP domains. We noticed that the NESmapper algorithm for predicting putative nuclear export signals (NES) identifies the Ta-sro1 peptide GQPVDSAVRKLLLE (247–260) as a likely NES with a score of 15.1 (Kosugi et al., 2014). The exchange of Val^250^ to Gly lowers the NESmapper score to 2.5 indicating that the two proteoforms could have different nuclear export rates. However, when we expressed green fluorescent protein (GFP)-tagged variants of the two Ta-SRO1 proteoforms in *Nicotiana benthamiana*, both proteins appeared entirely nuclear localized suggesting that at least steady-state transport kinetics over the nuclear envelope are not substantially different (Supplemental Figure S1).

## Ta-sro1 is catalytically inactive with respect to canonical ADP-ribosylation

**Figure 2:**
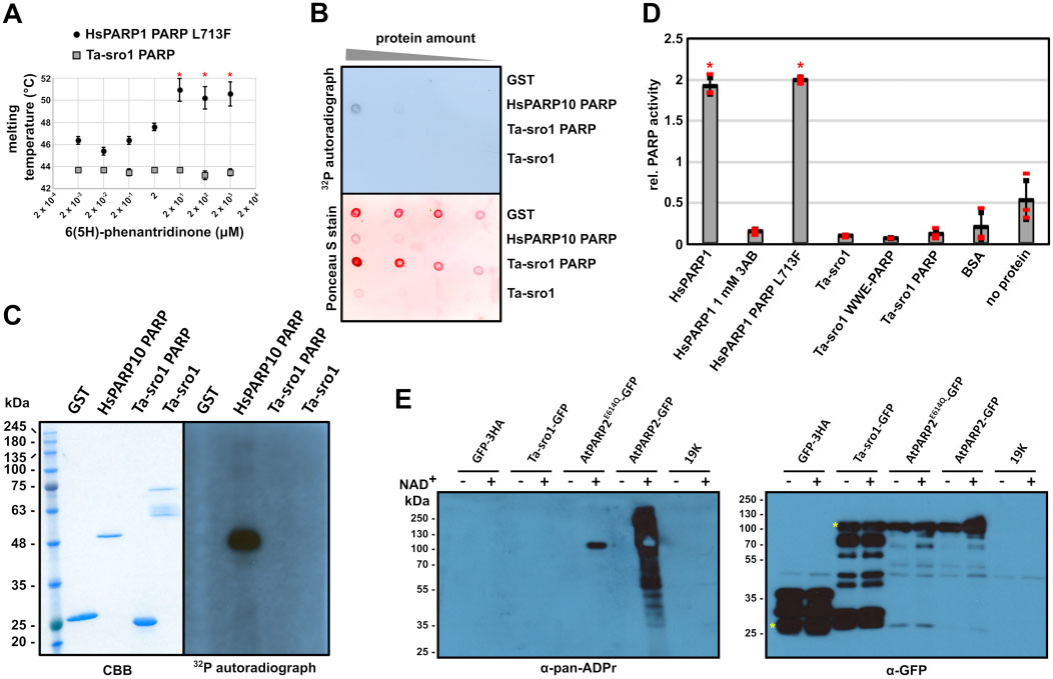
The Ta-sro1 PARP domain does not bind NAD^+^ and does not auto-ADP-ribosylate *in vitro* or in plant cells. A, Thermal stability profiles of the Ta-sro1 PARP domain and the HsPARP1 PARP domain determined by Differential Scanning Fluorimetry. Calculated melting temperatures were averaged from three independent experiments, each consisting of three technical replicates, *n* = 3. Melting temperatures are plotted against concentrations of the NAD^+^ analog 6(5H)-phenantridinone. Error bars represent standard deviations. Asterisks indicate significant differences compared to the lowest ligand concentration (one-way analysis of variance (ANOVA), Tukey’s honestly significant difference (HSD) test, *P* < 0.01). B, *In vitro*^32^P-NAD^+^-binding assay with full-length Ta-sro1 or the Ta-sro1 PARP domain, the HsPARP10 PARP domain, and GST. The Ponceau S stain indicates protein amounts. C, To assess the proteins from (B) for auto-ADP-ribosylation activity, they were incubated with [^32^P]-β-NAD^+^ at 30°C for 30 min. and the incorporated radioactivity was detected by exposure of the dried gel to X-ray film. CBB, Coomassie Brilliant Blue stain. D, Colorimetric PARP activity assay with histones as substrates. HsPARP1 refers to full-length HsPARP1 protein included in the assay kit as positive control. Error bars represent standard deviations, red bars show individual data points of technical replicates, *n* = 3. Asterisks indicate significant differences compared to the BSA control (one-way ANOVA, Tukey’s HSD, *P* < 0.01). Expression and purification procedures for proteins in (A–D) are described in Supplemental Methods. E, The indicated proteins were transiently expressed in *N. benthamiana* and extracted in the presence (+) or absence (−) of 300 µM β-NAD^+^. Proteins were purified via the GFP-tag and analyzed by α-GFP and α-pan-ADP-ribose binding reagent immunoblots. Leaves expressing only the viral silencing suppressor 19 K or GFP-3HA served as negative controls. Asterisks indicate the expected molecular weights of GFP-3HA and GFP-fusions of Ta-sro1 and AtPARP2. Results are representative of two (B and C) or three (D and E) independent experiments.

The observed structural divergence from the consensus motif at the active site of the Ta-sro1 PARP domain prompted us to test whether Ta-sro1 can indeed bind NAD^+^ and is able to perform ADP-ribosylation reactions. For mammalian PARP domains, thermal stabilization of the catalytic domain by small molecule inhibitors that mimic nicotinamide can serve as a proxy for NAD^+^ binding (Wahlberg et al., 2012). We determined the thermal stability of the Ta-sro1 PARP domain with increasing concentrations of the nicotinamide analog 6(5H)-phenanthridinone that was previously shown to stabilize ten catalytically active mammalian PARP domains (Wahlberg et al., 2012). We did not observe a thermal stabilization of the Ta-sro1 PARP domain by 6(5H)-phenanthridinone, even at concentrations in the millimolar range (Figure 2A). In contrast, the L713F variant of the HsPARP1 catalytic domain showed increased thermal stability (approximately +4°C) at 6(5H)-phenanthridinone concentrations above 2 µM (Figure 2A). We then performed *in vitro* binding assays with ^32^P-labeled NAD^+^ to test if the Ta-sro1 PARP domain can bind the presumed co-substrate. We also produced full-length Ta-sro1 protein using the expression plasmid from Liu et al. (2014) to test if flanking sequences of the PARP domain or other domains are required for NAD^+^ binding. As shown in Figure 2B, neither full-length Ta-sro1 nor the isolated PARP domain bound detectable amounts of ^32^P-NAD^+^. In contrast, the same assay detected ^32^P-NAD^+^ binding to HsPARP10 that was used as a positive control (Kleine et al., 2008). Many ADP-ribosyltransferases of the PARP family auto-ADP-ribosylate in the presence of NAD^+^ (Feng et al., 2015). Unlike the catalytic domain of HsPARP10 that attaches a single APD-ribose onto Glu residues in an intermolecular reaction (Kleine et al., 2008), neither the Ta-sro1 PARP domain nor the full-length protein showed auto-ADP-ribosylation *in vitro* when incubated with ^32^P-labeled NAD^+^ (Figure 2C). The postulated PARP activity of Ta-sro1 is based on a commercially available colorimetric assay (PARP Universal Colorimetric Assay Kit, Trevigen, Gaithersburg, MD, USA) that uses histones as substrates for ADP-ribosyltransferases. We repeated this assay according to the manufacturer’s manual (see “PARP Inhibitor Assay Protocol”) with 0.5 µg of protein per well and each reaction performed in triplicate for full-length Ta-sro1, the isolated PARP domain, and a protein fragment that also includes the N-terminal Trp-Trp-Glu (WWE) domain of the protein (Ta-sro1 WWE-PARP) (Figure 2D). Compared to the positive control included in the assay kit (full-length HsPARP1) and the variant HsPARP1 L713F catalytic domain produced in our laboratory, all three Ta-sro1 constructs were inactive with respect to PARP activity (Figure 2D). Values for relative PARP activities of the negative control BSA and a reaction without protein were in the same range as those of the Ta-sro1 constructs. The nicotinamide analog 3-aminobenzamide (3AB) inhibited ADP-ribosylation of histones by HsPARP1, demonstrating that the assay truly reflects ADP-ribosyltransferase activity (Figure 2D). Therefore, the standardized assay on which the previously reported Ta-sro1 PARP activity is based, is not reproducible under our conditions.

## Ta-sro1 does not auto-ADP-ribosylate when expressed in *N. benthamiana*

Several PARP enzymes require other proteins for catalytic activity or depend on a binding partner to modify specific amino acids (Yang et al., 2017; Suskiewicz et al., 2020). Given that the previously reported PARP activity of Ta-sro1 is based on an *in vitro* assay with recombinantly expressed protein, dependence on another plant factor to gain catalytic activity appears unlikely. Nevertheless, we assessed whether Ta-sro1 has mono- or poly-ADP-ribosyltransferase activity in plant cell extracts. We used transient Agrobacterium-mediated expression to produce Ta-sro1 and several control proteins as GFP fusions in *N. benthamiana*. We extracted the proteins in the absence or presence of 300 µM NAD^+^ and immunoprecipitated them using a GFP-nanobody coupled to magnetic beads (Chen et al., 2018). After separation by sodium dodecyl sulfate–polyacrylamide gel electrophoresis (SDS-PAGE), we probed soluble protein extracts with anti-pan-ADP-ribose binding reagent that binds to mono- and poly-(ADP-ribose) (Figure 2E). Ta-sro1-GFP did neither show signals for mono- nor for poly-ADP-ribosylation activity in this assay. In contrast, the canonical Arabidopsis PARP2 enzyme formed PAR chains in a NAD^+^-dependent manner. A PARP2 variant, in which the Glu residue that is essential for chain elongation is replaced by a Gln, produced a defined band of ∼100 kDa that could correspond to mono-ADP-ribosylated PARP2-GFP. These results suggest that even at relatively high concentrations of NAD^+^ and in presence of other plant co-factors that might be required for catalytic activity, Ta-sro1 does not exhibit mono- or poly-ADP-ribosyltransferase activity. Collectively, our reassessment of the proposed Ta-sro1 PARP activity revealed that the previously reported catalytic activity could not be reproduced under our conditions and with appropriate controls. Therefore, the elevated tolerance of wheat cultivar SR3 to high salinity and oxidative stress conditions cannot be explained by an altered PARP activity of the Ta-sro1 proteoform and other possible mechanisms need to be considered. SR3 plants exhibit higher H_2_O_2_ levels under salinity stress but also under control conditions (Liu et al., 2014). Given the role of reactive oxygen species in signal transduction and cell-to-cell communication in response to salinity stress (Evans et al., 2016), the altered redox homeostasis of wheat cultivar SR3 may underlie its enhanced salt tolerance phenotype. Arabidopsis RCD1 lacks ADP-ribosyltransferase activity and *rcd1* mutants also exhibit an altered sensitivity to reactive oxygen species (Shapiguzov et al., 2019). This suggests that redox imbalances in *sro* mutants are not necessarily caused by an altered ADP-ribosyltransferase activity of SRO proteins, but more likely relate to their function as transcriptional co-regulators.

## Are plant SRO proteins noncanonical ADP-ribosyltransferases?

**Figure 3:**
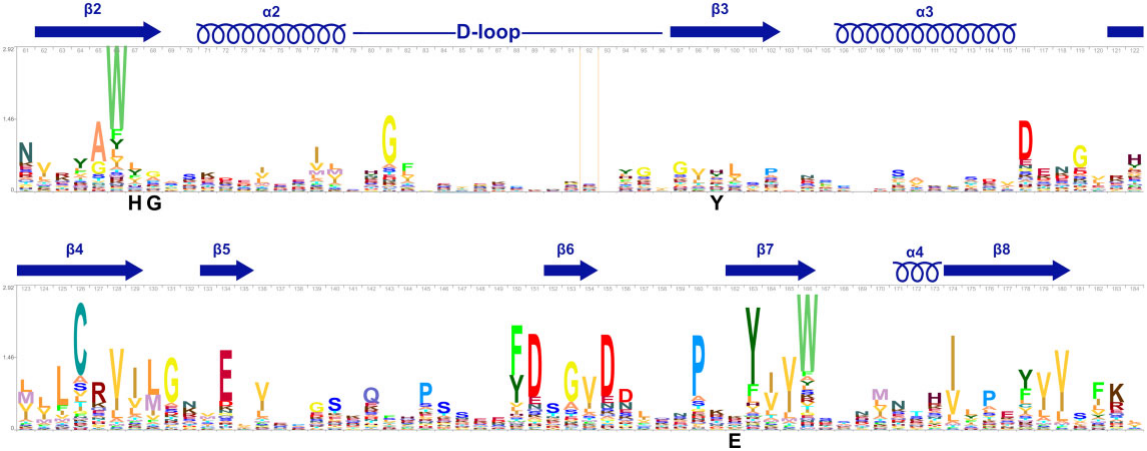
The His–Tyr–Glu triad is not conserved in the SRO protein family. SRO proteins were identified using JACKHMMER (Potter et al., 2018) with the Ta-sro1 PARP domain as a query and the following settings: HmmerWeb version 2.41.2, database Reference proteomes, restricted to *Viridiplantae* (taxid:33090), E-values Sequence = 0.0001, Hit = 0.0003. The sequence conservation logo was generated with Skylign (Skylign.org by Jody Clements, Travis Wheeler & Robert Finn, Interactive logos for alignments and profile HMMs, http://skylign.org/, CC BY 3.0 license) and corresponds to Ta-sro1 amino acids 306–429. The positions of the conserved His, Gly, Tyr, and Glu are indicated. Conserved secondary structure elements are depicted above the sequence logo.

The comparably low number of canonical PARPs in plants, paired with the structural conservation of PARP(-like) domains in SRO proteins, have fueled the hypothesis that SROs may represent an additional plant-specific family of noncanonical ADP-ribosyltransferases (Lamb et al., 2012; Kong et al., 2021). Given that a His–Tyr–[Glu/Asp/Gln] triad is conserved in ADP-ribosyltransferases from diphtheria toxin to mammalian and plant PARPs, we explored the level of conservation of the corresponding residues in plant SRO proteins. We used JACKHMMER (Potter et al., 2018) and the Ta-sro1 PARP domain as a query to identify sequence-related PARP domains within the *Viridiplantae*. This search identified 1,399 homologs until first members of canonical PARPs started to appear in the second iteration. The sequence logo in Figure 3 is representative of 1,084 *bona fide* SRO PARP domains from proteins with a (WWE-)PARP-RST domain structure, 312 homologs without additional annotated domains, and three proteins with a domain structure representative of canonical PARPs. As shown in Figure 3, in SRO PARP domains, there is no strong conservation of the His and Tyr that coordinate NAD^+^ binding and the same holds true for the Glu residue required for polymerase activity. Notably, in a subset of 33 PARP domains from 14 different plant families the His and Tyr residues are conserved and in all of these the His is followed by a Gly (Supplemental Data Set 1). In contrast, there is no conservation of the Glu in these SRO family members and the equivalent position is often taken by a Trp or Arg. This indicates that structural features of the NAD^+^-binding site are conserved in a few SRO family members and consequently these proteins might mono-ADP-ribosylate their substrates by substrate-assisted catalysis. From an evolutionary perspective, the plant SRO protein family therefore represents a valuable resource of natural variation in the otherwise highly conserved PARP domain. As reported by Kong et al. (2021), at least one SRO protein retained the ability to catalyze mono-ADP-ribosylation reactions. In Arabidopsis SRO2, the positions of the His and Tyr residues are swapped suggesting that certain deviations from the consensus motif are compatible with ADP-ribosyltransferase activity. The position of the conserved Gly is taken by Ala^119^ in SRO2. Unlike the bulky pyrrolidine ring of the Pro residues in the Arabidopsis RCD1 and Ta-sro1 structures at this position, the smaller Ala side chain is less likely to interfere with accommodation of the nicotinamide moiety and the Ala backbone retains the capacity to form two hydrogen bonds with the co-substrate. It will be informative to correlate the level of divergence in key residues of the NAD^+^-binding site with ADP-ribosyltransferase activity in other members of the SRO family. Future analyses should further explore how SRO proteins influence transcription factor activity in abiotic and biotic stress responses and address the question of why PARP domains are strictly conserved in this group of transcriptional co-regulators. However, we argue that, given the nonconservation of the His–Tyr–Glu triad in the majority of SRO proteins, the biochemical basis for reported noncanonical ADP-ribosyltransferase activities should be investigated to substantiate their mode of catalytic activity. Many PARP domains can be readily produced as soluble proteins in *E. coli* and assays to test for NAD^+^ (analog) binding, as well as mono- and/or poly-ADP-ribosyltransferase activity are outlined in this letter and well documented for mammalian PARPs (Kleine et al., 2008; Wahlberg et al., 2012; Glumoff et al., 2022). Analyses of noncanonical plant ADP-ribosyltransferases should include site-directed mutagenesis of residues predicted to coordinate NAD^+^ binding combined with enzyme assays and, where feasible, complementation studies of the respective plant mutant phenotypes as well as evidence for ADP-ribosylation of substrates by protein mass spectrometry.

## Supplemental data

The following materials are available in the online version of this article.

**Supplemental Figure S1.** The Ta-sro1 and Ta-SRO1 proteoforms do not show differences in subcellular localization when transiently expressed in *N. benthamiana*.

**Supplemental Table S1.** X-ray data collection, refinement, and validation statistics of the Ta-sro1 PARP domain structure.

**Supplemental Table S2.** Statistical analysis of experimental data presented in Figure 2A.

**Supplemental Table S3.** Statistical analysis of experimental data presented in Figure 2D.

**Supplemental Table S4.** List of oligonucleotides used in this work.

**Supplemental Methods.** Experimental procedures for protein expression, purification, and crystallization as well as the activity and binding assays.

**Supplemental File S1.** Protein Data Bank X-Ray Validation Report.

**Supplemental Data Set 1.** The His, Gly, and Tyr residues that coordinate NAD^+^ binding are conserved in several plant PARP domains beyond canonical PARPs.

## Supplementary Material

koac261_Supplementary_Data

## Notes

K.F., S.H., R.D.M., B.L., and L.W. conceived and designed experiments. S.V., K.F., S.H., R.D.M., R.L., B.L., and L.W. carried out experiments. S.V., K.F., S.H., B.L., and L.W. analyzed the data. K.F., B.L., and L.W. wrote the manuscript. All authors reviewed and approved the submitted manuscript.

The author responsible for distribution of materials integral to the findings presented in this article in accordance with the policy described in the Instructions for Authors (https://academic.oup.com/plcell) is: Lennart Wirthmueller (lennart.wirthmueller@ipb-halle.de).
