# Supplementary material for: The superior salinity tolerance of bread wheat cultivar Shanrong No. 3 is unlikely to be caused by elevated Ta-sro1 poly-(ADP-ribose) polymerase activity
Source: Plant Cell. 2022 Aug 18;34(11):4130–7. doi: 10.1093/plcell/koac261 (PMC9614482; doi:10.1093/plcell/koac261)
Supplement: koac261_Supplementary_Data [file koac261_supplementary_data.zip › koac261_Supplementary_Data/7plq_full_validation.pdf]

Full wwPDB X-ray Structure Validation Report (i)

Oct 21, 2021 – 04:15 pm BST

PDB ID	:	7PLQ
Title	:	Crystal structure of the PARP domain of wheat SRO1
Authors	:	Wirthmueller, L.; Loll, B.
Deposited on	:	2021-09-01
Resolution	:	2.13 Å(reported)

This is a Full wwPDB X-ray Structure Validation Report for a publicly released PDB entry.

We welcome your comments at *validation@mail.wwpdb.org*
A user guide is available at
https://www.wwpdb.org/validation/2017/XrayValidationReportHelp
with specific help available everywhere you see the (i) symbol.

The following versions of software and data (see references (i)) were used in the production of this report:

MolProbity	:	4.02b-467
Mogul	:	1.8.5 (274361), CSD as541be (2020)
Xtriage (Phenix)	:	1.13
EDS	:	2.23.2
Percentile statistics	:	20191225.v01 (using entries in the PDB archive December 25th 2019)
Refmac	:	5.8.0267
CCP4	:	7.1.010 (Gargrove)
Ideal geometry (proteins)	:	Engh & Huber (2001)
Ideal geometry (DNA, RNA)	:	Parkinson et al. (1996)
Validation Pipeline (wwPDB-VP)	:	2.23.2

1 Overall quality at a glance (i)

The following experimental techniques were used to determine the structure:
X-RAY DIFFRACTION

The reported resolution of this entry is 2.13 Å.

Percentile scores (ranging between 0-100) for global validation metrics of the entry are shown in the following graphic. The table shows the number of entries on which the scores are based.

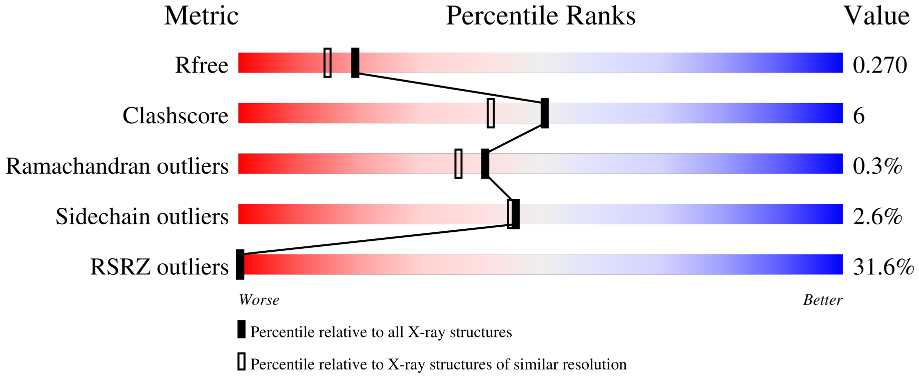

Metric	Whole archive (#Entries)	Similar resolution (#Entries, resolution range(Å))
R_{free}	130704	2523 (2.16-2.12)
Clashscore	141614	2653 (2.16-2.12)
Ramachandran outliers	138981	2618 (2.16-2.12)
Sidechain outliers	138945	2617 (2.16-2.12)
RSRZ outliers	127900	2485 (2.16-2.12)

The table below summarises the geometric issues observed across the polymeric chains and their fit to the electron density. The red, orange, yellow and green segments of the lower bar indicate the fraction of residues that contain outliers for >=3, 2, 1 and 0 types of geometric quality criteria respectively. A grey segment represents the fraction of residues that are not modelled. The numeric value for each fraction is indicated below the corresponding segment, with a dot representing fractions <=5% The upper red bar (where present) indicates the fraction of residues that have poor fit to the electron density. The numeric value is given above the bar.

Mol	Chain	Length	Quality of chain
1	A	191	29%<br>76% 16% • 7%
1	B	191	28%<br>82% 14% • •

2 Entry composition (i)

There are 6 unique types of molecules in this entry. The entry contains 3000 atoms, of which 0 are hydrogens and 0 are deuteriums.

In the tables below, the ZeroOcc column contains the number of atoms modelled with zero occupancy, the AltConf column contains the number of residues with at least one atom in alternate conformation and the Trace column contains the number of residues modelled with at most 2 atoms.

- Molecule 1 is a protein called SIMILAR TO RCD1 (SRO1).

Mol	Chain	Residues	Atoms						ZeroOcc	AltConf	Trace
1	A	178	Total 1421	C 895	N 251	O 261	S 4	Se 10	0	2	0
1	B	183	Total 1463	C 919	N 259	O 271	S 4	Se 10	0	3	0

There are 4 discrepancies between the modelled and reference sequences:

Chain	Residue	Modelled	Actual	Comment	Reference
A	244	GLY	-	expression tag	UNP A0A3B6LXD6
A	245	PRO	-	expression tag	UNP A0A3B6LXD6
B	244	GLY	-	expression tag	UNP A0A3B6LXD6
B	245	PRO	-	expression tag	UNP A0A3B6LXD6

- Molecule 2 is SULFATE ION (three-letter code: SO4) (formula: O_4S).

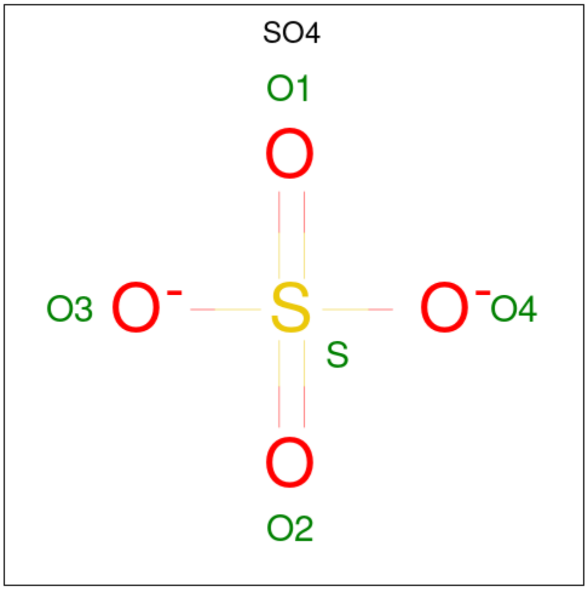

Mol	Chain	Residues	Atoms	ZeroOcc	AltConf
2	A	1	Total O S<br>5 4 1	0	0
2	B	1	Total O S<br>5 4 1	0	0

- Molecule 3 is GLYCEROL (three-letter code: GOL) (formula: $C_3H_8O_3$).

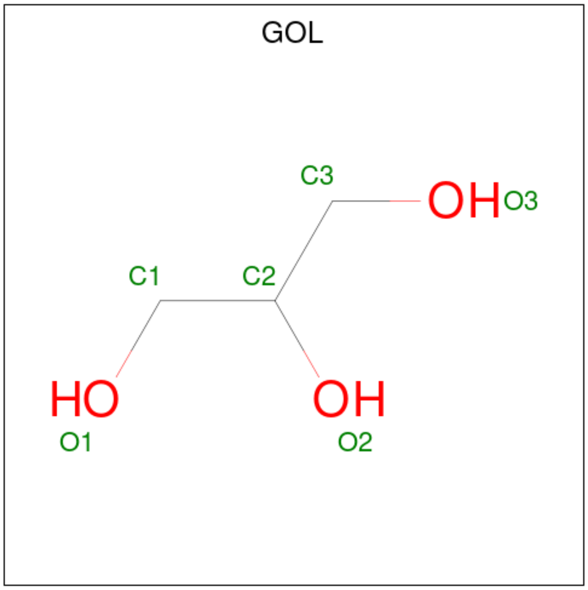

Mol	Chain	Residues	Atoms	ZeroOcc	AltConf
3	A	1	Total C O<br>6 3 3	0	0

- Molecule 4 is 1-ETHOXY-2-(2-ETHOXYETHOXY)ETHANE (three-letter code: P4G) (formula: $C_8H_{18}O_3$).

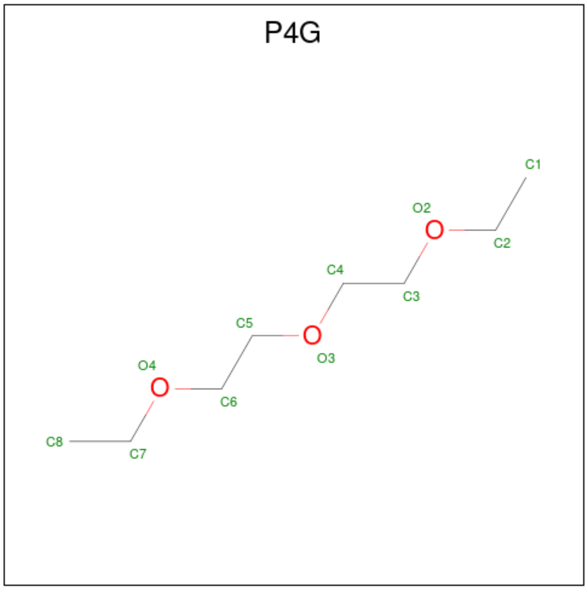

Mol	Chain	Residues	Atoms	ZeroOcc	AltConf
4	B	1	Total C O<br>9 6 3	0	0

- Molecule 5 is 1,2-ETHANEDIOL (three-letter code: EDO) (formula: $C_2H_6O_2$).

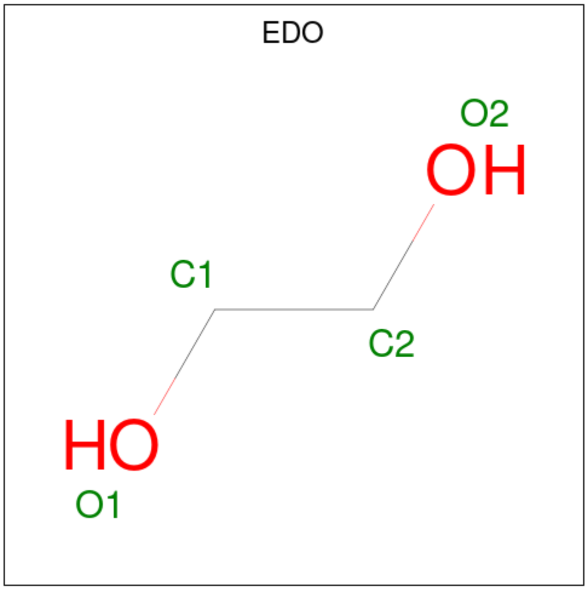

Mol	Chain	Residues	Atoms	ZeroOcc	AltConf
5	B	1	Total C O<br>4 2 2	0	0
5	B	1	Total C O<br>4 2 2	0	0

- Molecule 6 is water.

Mol	Chain	Residues	Atoms	ZeroOcc	AltConf
6	A	44	Total O<br>44 44	0	4
6	B	39	Total O<br>39 39	0	0

3 Residue-property plots (i)

These plots are drawn for all protein, RNA, DNA and oligosaccharide chains in the entry. The first graphic for a chain summarises the proportions of the various outlier classes displayed in the second graphic. The second graphic shows the sequence view annotated by issues in geometry and electron density. Residues are color-coded according to the number of geometric quality criteria for which they contain at least one outlier: green = 0, yellow = 1, orange = 2 and red = 3 or more. A red dot above a residue indicates a poor fit to the electron density (RSRZ > 2). Stretches of 2 or more consecutive residues without any outlier are shown as a green connector. Residues present in the sample, but not in the model, are shown in grey.

- Molecule 1: SIMILAR TO RCD1 (SRO1)

Chain A:

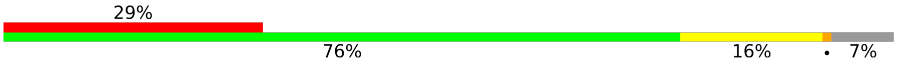

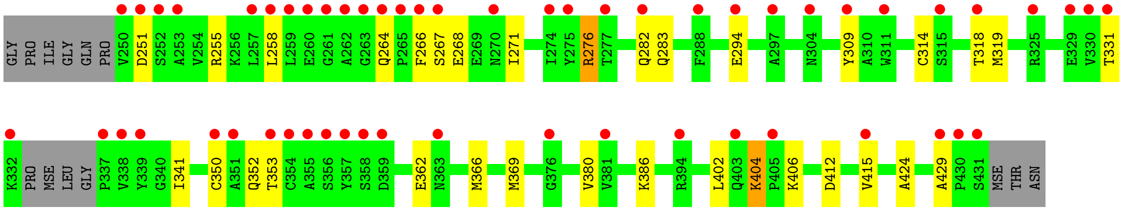

- Molecule 1: SIMILAR TO RCD1 (SRO1)

Chain B:

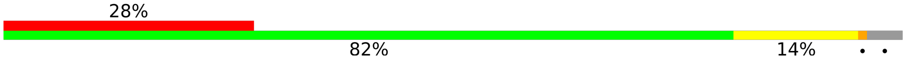

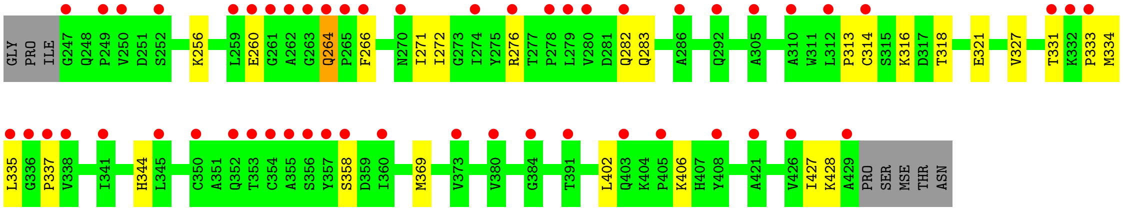

4 Data and refinement statistics ⓘ

Property	Value	Source
Space group	C 1 2 1	Depositor
Cell constants<br>a, b, c, α, β, γ	78.51Å 34.37Å 132.58Å<br>90.00° 101.22° 90.00°	Depositor
Resolution (Å)	43.35 – 2.13<br>43.35 – 2.13	Depositor<br>EDS
% Data completeness<br>(in resolution range)	97.6 (43.35-2.13)<br>97.7 (43.35-2.13)	Depositor<br>EDS
R_{merge}	(Not available)	Depositor
R_{sym}	(Not available)	Depositor
$< I/\sigma(I) >$ [1]	0.99 (at 2.12Å)	Xtriage
Refinement program	PHENIX 1.19.1_4122	Depositor
R, R_{free}	0.214 , 0.266<br>0.220 , 0.270	Depositor<br>DCC
R_{free} test set	974 reflections (5.00%)	wwPDB-VP
Wilson B-factor (Å^2)	40.4	Xtriage
Anisotropy	0.246	Xtriage
Bulk solvent k_{sol}(e/Å^3), B_{sol}(Å^2)	(Not available) , (Not available)	EDS
L-test for twinning[2]	$< \|L\| > = 0.47$, $< L^2 > = 0.30$	Xtriage
Estimated twinning fraction	No twinning to report.	Xtriage
F_o,F_c correlation	0.94	EDS
Total number of atoms	3000	wwPDB-VP
Average B, all atoms (Å^2)	52.0	wwPDB-VP

Xtriage's analysis on translational NCS is as follows: *The largest off-origin peak in the Patterson function is 15.53% of the height of the origin peak. No significant pseudotranslation is detected.*

[1]Intensities estimated from amplitudes.
[2]Theoretical values of $< |L| >$, $< L^2 >$ for acentric reflections are 0.5, 0.333 respectively for untwinned datasets, and 0.375, 0.2 for perfectly twinned datasets.

5 Model quality (i)

5.1 Standard geometry (i)

Bond lengths and bond angles in the following residue types are not validated in this section: P4G, GOL, SO4, EDO

The Z score for a bond length (or angle) is the number of standard deviations the observed value is removed from the expected value. A bond length (or angle) with $|Z| > 5$ is considered an outlier worth inspection. RMSZ is the root-mean-square of all Z scores of the bond lengths (or angles).

Mol	Chain	Bond lengths		Bond angles	
		RMSZ	#|Z| >5	RMSZ	#|Z| >5
1	A	0.29	0/1440	0.55	0/1929
1	B	0.30	0/1483	0.57	0/1988
All	All	0.30	0/2923	0.56	0/3917

Chiral center outliers are detected by calculating the chiral volume of a chiral center and verifying if the center is modelled as a planar moiety or with the opposite hand.A planarity outlier is detected by checking planarity of atoms in a peptide group, atoms in a mainchain group or atoms of a sidechain that are expected to be planar.

Mol	Chain	#Chirality outliers	#Planarity outliers
1	B	0	1

There are no bond length outliers.

There are no bond angle outliers.

There are no chirality outliers.

All (1) planarity outliers are listed below:

Mol	Chain	Res	Type	Group
1	B	264	GLN	Peptide

5.2 Too-close contacts (i)

In the following table, the Non-H and H(model) columns list the number of non-hydrogen atoms and hydrogen atoms in the chain respectively. The H(added) column lists the number of hydrogen atoms added and optimized by MolProbity. The Clashes column lists the number of clashes within the asymmetric unit, whereas Symm-Clashes lists symmetry-related clashes.

Mol	Chain	Non-H	H(model)	H(added)	Clashes	Symm-Clashes
1	A	1421	0	1411	19	0
1	B	1463	0	1445	15	0
2	A	5	0	0	1	0
2	B	5	0	0	0	0
3	A	6	0	8	0	0
4	B	9	0	13	1	0
5	B	8	0	12	1	0
6	A	44	0	0	1	0
6	B	39	0	0	0	0
All	All	3000	0	2889	33	0

The all-atom clashscore is defined as the number of clashes found per 1000 atoms (including hydrogen atoms). The all-atom clashscore for this structure is 6.

All (33) close contacts within the same asymmetric unit are listed below, sorted by their clash magnitude.

Atom-1	Atom-2	Interatomic distance (Å)	Clash overlap (Å)
1:A:251:ASP:OD1	1:A:276:ARG:NH2	2.23	0.70
1:A:294:GLU:OE1	6:A:601:HOH:O	2.11	0.69
1:A:380:VAL:HG23	1:A:402:LEU:HD23	1.80	0.63
1:B:333:PRO:O	1:B:335:LEU:N	2.38	0.57
1:A:282:GLN:HG3	1:A:283:GLN:HG3	1.86	0.56
1:A:314:CYS:SG	1:A:369:MSE:HG3	2.46	0.56
1:B:318:THR:HG21	1:B:331:THR:HG22	1.89	0.55
1:A:404:LYS:HE3	1:A:406:LYS:HE3	1.89	0.53
1:B:256:LYS:NZ	1:B:260:GLU:OE1	2.33	0.52
1:A:366:MSE:HE2	1:A:429:ALA:HA	1.92	0.52
1:A:255:ARG:NE	1:A:268:GLU:OE2	2.31	0.51
1:B:337:PRO:HB2	1:B:344:HIS:CD2	2.46	0.50
1:A:318:THR:HG21	1:A:331:THR:HG23	1.94	0.50
1:A:271:ILE:O	1:B:316:LYS:NZ	2.33	0.49
1:B:314:CYS:SG	1:B:369:MSE:HG3	2.53	0.49
1:B:313:PRO:HB3	1:B:358[A]:SER:HB3	1.95	0.49
1:A:380:VAL:CG2	1:A:402:LEU:HD23	2.44	0.48
1:B:282:GLN:HG3	1:B:283:GLN:HG3	1.96	0.47
1:B:406:LYS:HA	1:B:406:LYS:HD3	1.75	0.47
1:A:341:ILE:HD13	1:A:386:LYS:HA	1.97	0.47
1:B:321:GLU:HB3	1:B:327:VAL:HG23	1.97	0.46
1:A:350:CYS:HB3	1:A:353:THR:HB	1.96	0.46
1:B:271:ILE:HA	1:B:427:ILE:HD13	1.99	0.45
1:A:264:GLN:HB2	1:A:352:GLN:HG3	1.99	0.44
1:A:319:MSE:HE2	1:A:424:ALA:HB1	2.00	0.44

Continued on next page...

Continued from previous page...

Atom-1	Atom-2	Interatomic distance (Å)	Clash overlap (Å)
1:A:412:ASP:O	1:A:415[B]:VAL:HG22	2.19	0.43
1:B:272:ILE:HD11	1:B:428:LYS:HB2	2.00	0.43
1:A:309:TYR:HB2	2:A:501:SO4:O1	2.19	0.43
1:A:406:LYS:HD3	1:A:406:LYS:HA	1.60	0.43
1:B:282:GLN:HA	5:B:502:EDO:H22	2.00	0.43
1:B:402:LEU:HD12	4:B:501:P4G:H51	2.01	0.42
1:A:258:LEU:HD23	1:A:258:LEU:HA	1.91	0.41
1:B:402:LEU:HD23	1:B:402:LEU:HA	1.96	0.41

There are no symmetry-related clashes.

5.3 Torsion angles (i)

5.3.1 Protein backbone (i)

In the following table, the Percentiles column shows the percent Ramachandran outliers of the chain as a percentile score with respect to all X-ray entries followed by that with respect to entries of similar resolution.

The Analysed column shows the number of residues for which the backbone conformation was analysed, and the total number of residues.

Mol	Chain	Analysed	Favoured	Allowed	Outliers	Percentiles	
1	A	176/191 (92%)	174 (99%)	2 (1%)	0	100	100
1	B	184/191 (96%)	177 (96%)	6 (3%)	1 (0%)	29	22
All	All	360/382 (94%)	351 (98%)	8 (2%)	1 (0%)	41	36

All (1) Ramachandran outliers are listed below:

Mol	Chain	Res	Type
1	B	334	MSE

5.3.2 Protein sidechains (i)

In the following table, the Percentiles column shows the percent sidechain outliers of the chain as a percentile score with respect to all X-ray entries followed by that with respect to entries of similar resolution.

The Analysed column shows the number of residues for which the sidechain conformation was analysed, and the total number of residues.

Mol	Chain	Analysed	Rotameric	Outliers	Percentiles	
1	A	154/151 (102%)	149 (97%)	5 (3%)	39	37
1	B	158/151 (105%)	155 (98%)	3 (2%)	57	59
All	All	312/302 (103%)	304 (97%)	8 (3%)	46	45

All (8) residues with a non-rotameric sidechain are listed below:

Mol	Chain	Res	Type
1	A	266	PHE
1	A	267	SER
1	A	276	ARG
1	A	362	GLU
1	A	404	LYS
1	B	264	GLN
1	B	266	PHE
1	B	276	ARG

Sometimes sidechains can be flipped to improve hydrogen bonding and reduce clashes. There are no such sidechains identified.

5.3.3 RNA (i)

There are no RNA molecules in this entry.

5.4 Non-standard residues in protein, DNA, RNA chains (i)

There are no non-standard protein/DNA/RNA residues in this entry.

5.5 Carbohydrates (i)

There are no monosaccharides in this entry.

5.6 Ligand geometry (i)

6 ligands are modelled in this entry.

In the following table, the Counts columns list the number of bonds (or angles) for which Mogul statistics could be retrieved, the number of bonds (or angles) that are observed in the model and the number of bonds (or angles) that are defined in the Chemical Component Dictionary. The Link column lists molecule types, if any, to which the group is linked. The Z score for a bond length (or angle) is the number of standard deviations the observed value is removed from the

expected value. A bond length (or angle) with $|Z| > 2$ is considered an outlier worth inspection. RMSZ is the root-mean-square of all Z scores of the bond lengths (or angles).

Mol	Type	Chain	Res	Link	Bond lengths			Bond angles		
					Counts	RMSZ	#|Z| > 2	Counts	RMSZ	#|Z| > 2
4	P4G	B	501	-	8,8,10	0.54	0	7,7,9	0.28	0
2	SO4	B	504	-	4,4,4	0.14	0	6,6,6	0.13	0
5	EDO	B	502	-	3,3,3	0.46	0	2,2,2	0.35	0
5	EDO	B	503	-	3,3,3	0.50	0	2,2,2	0.32	0
3	GOL	A	502	-	5,5,5	0.90	0	5,5,5	1.01	0
2	SO4	A	501	-	4,4,4	0.12	0	6,6,6	0.19	0

In the following table, the Chirals column lists the number of chiral outliers, the number of chiral centers analysed, the number of these observed in the model and the number defined in the Chemical Component Dictionary. Similar counts are reported in the Torsion and Rings columns. '-' means no outliers of that kind were identified.

Mol	Type	Chain	Res	Link	Chirals	Torsions	Rings
5	EDO	B	503	-	-	1/1/1/1	-
3	GOL	A	502	-	-	2/4/4/4	-
5	EDO	B	502	-	-	0/1/1/1	-
4	P4G	B	501	-	-	5/6/6/8	-

There are no bond length outliers.

There are no bond angle outliers.

There are no chirality outliers.

All (8) torsion outliers are listed below:

Mol	Chain	Res	Type	Atoms
3	A	502	GOL	C1-C2-C3-O3
3	A	502	GOL	O2-C2-C3-O3
4	B	501	P4G	O3-C5-C6-O4
4	B	501	P4G	C4-C3-O2-C2
4	B	501	P4G	C6-C5-O3-C4
4	B	501	P4G	C1-C2-O2-C3
5	B	503	EDO	O1-C1-C2-O2
4	B	501	P4G	O2-C3-C4-O3

There are no ring outliers.

3 monomers are involved in 3 short contacts:

Mol	Chain	Res	Type	Clashes	Symm-Clashes
4	B	501	P4G	1	0
5	B	502	EDO	1	0
2	A	501	SO4	1	0

5.7 Other polymers (i)

There are no such residues in this entry.

5.8 Polymer linkage issues (i)

There are no chain breaks in this entry.

6 Fit of model and data (i)

6.1 Protein, DNA and RNA chains (i)

In the following table, the column labelled '#RSRZ> 2' contains the number (and percentage) of RSRZ outliers, followed by percent RSRZ outliers for the chain as percentile scores relative to all X-ray entries and entries of similar resolution. The OWAB column contains the minimum, median, 95th percentile and maximum values of the occupancy-weighted average B-factor per residue. The column labelled 'Q< 0.9' lists the number of (and percentage) of residues with an average occupancy less than 0.9.

Mol	Chain	Analysed	<RSRZ>	#RSRZ>2			OWAB(Å^2)	Q<0.9
1	A	169/191 (88%)	2.06	55 (32%)	0	0	31, 47, 103, 174	0
1	B	173/191 (90%)	1.94	53 (30%)	0	0	26, 44, 105, 132	0
All	All	342/382 (89%)	2.00	108 (31%)	0	0	26, 45, 105, 174	0

All (108) RSRZ outliers are listed below:

Mol	Chain	Res	Type	RSRZ
1	A	357	TYR	15.6
1	A	431	SER	13.8
1	B	357	TYR	11.0
1	B	260	GLU	10.5
1	B	358[A]	SER	10.3
1	A	356	SER	9.3
1	A	263	GLY	8.6
1	A	264	GLN	7.7
1	B	263	GLY	7.7
1	B	336	GLY	7.6
1	A	331	THR	7.0
1	A	355	ALA	7.0
1	B	355	ALA	6.8
1	A	261	GLY	6.6
1	A	260	GLU	6.5
1	B	265	PRO	6.4
1	A	353	THR	6.2
1	B	335	LEU	5.8
1	B	259	LEU	5.8
1	A	270	ASN	5.7
1	A	330	VAL	5.6
1	B	429	ALA	5.1
1	B	331	THR	5.1
1	B	261	GLY	4.9

Continued on next page...

Continued from previous page...

Mol	Chain	Res	Type	RSRZ
1	A	430	PRO	4.8
1	B	264	GLN	4.6
1	B	266	PHE	4.5
1	A	259	LEU	4.3
1	A	282	GLN	4.3
1	A	358	SER	4.2
1	A	266	PHE	4.1
1	B	354	CYS	4.1
1	A	337	PRO	4.0
1	B	250	VAL	4.0
1	A	359	ASP	3.9
1	B	352	GLN	3.9
1	B	353	THR	3.9
1	A	318	THR	3.9
1	B	247	GLY	3.8
1	A	257	LEU	3.8
1	A	332	LYS	3.8
1	A	376	GLY	3.8
1	A	262	ALA	3.7
1	A	250	VAL	3.7
1	B	338	VAL	3.7
1	A	350	CYS	3.6
1	B	356	SER	3.6
1	B	333	PRO	3.6
1	A	265	PRO	3.5
1	B	282	GLN	3.4
1	A	251	ASP	3.3
1	A	252	SER	3.3
1	B	305	ALA	3.2
1	B	314	CYS	3.2
1	A	311	TRP	3.1
1	B	249	PRO	3.0
1	B	360	ILE	3.0
1	A	274	ILE	2.9
1	A	429	ALA	2.9
1	A	253	ALA	2.8
1	B	350	CYS	2.8
1	B	252	SER	2.8
1	B	337	PRO	2.7
1	A	339	TYR	2.7
1	A	338	VAL	2.7
1	B	262	ALA	2.7

Continued on next page...

Continued from previous page...

Mol	Chain	Res	Type	RSRZ
1	A	309	TYR	2.6
1	B	332	LYS	2.6
1	A	258	LEU	2.6
1	A	415[A]	VAL	2.6
1	A	288	PHE	2.5
1	A	294	GLU	2.5
1	A	304	ASN	2.5
1	B	278	PRO	2.5
1	B	380	VAL	2.5
1	B	408	TYR	2.4
1	A	394	ARG	2.4
1	A	363	ASN	2.4
1	A	315	SER	2.3
1	B	345	LEU	2.3
1	A	405	PRO	2.3
1	B	426	VAL	2.3
1	B	405	PRO	2.3
1	B	286	ALA	2.3
1	B	373	VAL	2.3
1	A	277	THR	2.3
1	B	292	GLN	2.3
1	B	276	ARG	2.2
1	B	270	ASN	2.2
1	B	274	ILE	2.2
1	B	341	ILE	2.2
1	B	403[A]	GLN	2.2
1	A	381	VAL	2.2
1	B	384	GLY	2.2
1	B	421	ALA	2.2
1	B	391	THR	2.2
1	B	280	VAL	2.1
1	A	351	ALA	2.1
1	A	354	CYS	2.1
1	B	279	LEU	2.1
1	A	275	TYR	2.1
1	A	325	ARG	2.1
1	B	312	LEU	2.1
1	B	310	ALA	2.1
1	A	329	GLU	2.1
1	A	267	SER	2.0
1	A	403	GLN	2.0
1	A	297	ALA	2.0

6.2 Non-standard residues in protein, DNA, RNA chains (i)

There are no non-standard protein/DNA/RNA residues in this entry.

6.3 Carbohydrates (i)

There are no monosaccharides in this entry.

6.4 Ligands (i)

In the following table, the Atoms column lists the number of modelled atoms in the group and the number defined in the chemical component dictionary. The B-factors column lists the minimum, median, 95th percentile and maximum values of B factors of atoms in the group. The column labelled 'Q< 0.9' lists the number of atoms with occupancy less than 0.9.

Mol	Type	Chain	Res	Atoms	RSCC	RSR	B-factors(Å^2)	Q<0.9
3	GOL	A	502	6/6	0.62	0.23	57,71,80,80	0
5	EDO	B	503	4/4	0.71	0.26	36,36,38,52	0
4	P4G	B	501	9/11	0.75	0.23	46,60,67,68	0
5	EDO	B	502	4/4	0.82	0.23	50,56,60,63	0
2	SO4	A	501	5/5	0.97	0.17	36,39,47,50	0
2	SO4	B	504	5/5	0.97	0.18	40,41,45,55	0

6.5 Other polymers (i)

There are no such residues in this entry.
