# Supplementary material for: The superior salinity tolerance of bread wheat cultivar Shanrong No. 3 is unlikely to be caused by elevated Ta-sro1 poly-(ADP-ribose) polymerase activity
Source: Plant Cell. 2022 Aug 18;34(11):4130–7. doi: 10.1093/plcell/koac261 (PMC9614482; doi:10.1093/plcell/koac261)
Supplement: koac261_Supplementary_Data [file koac261_supplementary_data.zip › koac261_Supplementary_Data/TPC2022LTE00184DR1_Supplemental_Data.pdf]

Supplemental Data. Vogt et al. (2022) The superior salinity tolerance of bread wheat cultivar Shanrong No. 3 is unlikely to be caused by elevated Ta-sro1 poly-(ADP-ribose) polymerase activity.

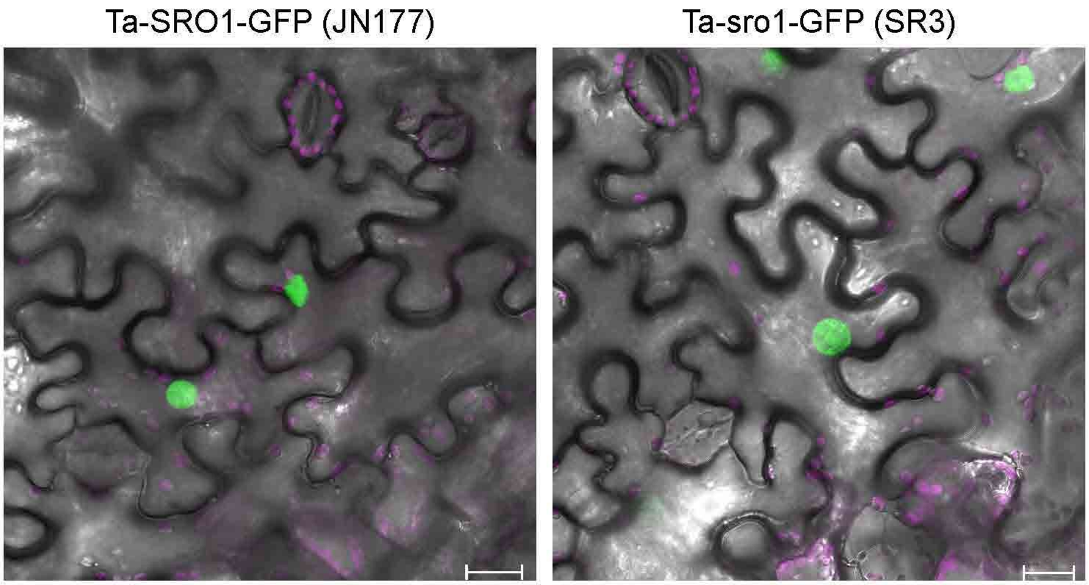

Supplemental Figure S1. Supports Figure 1. The Ta-sro1 and Ta-SRO1 proteoforms do not show differences in subcellular localization when transiently expressed in *N. benthamiana*. GFP-tagged versions of the two proteoforms were analyzed by confocal microscopy. The GFP signal is false-colored green, purple color shows chlorophyll fluorescence from chloroplasts. Scale bars = 20 μm. Leaf discs excised from *N. benthamiana* were mounted on microscope slides in 50% (v/v) glycerol and the subcellular localization of GFP-tagged proteins was analyzed using a Zeiss LSM 780 confocal microscope. Excitation wavelength and emission collection windows were 488/493-545 nm for GFP and 561/687-730 nm for chlorophyll. Images were acquired and analyzed using Zeiss Zen software. The images are representative of five independent leaf sections (n=5) per proteoform monitored in a single experiment.

Supplemental Table S1. X-ray data collection, refinement, and validation statistics of the Ta-sro1 PARP domain structure.

**Data collection**	
Beamline	BESSYII-14.2
Wavelength (Å)	0.97999
Space group	*C*2
Unit cell parameters	
a, b, c (Å)	78.51, 34.37, 132.58
α, β, γ (°)	90, 101.23, 90
Unique reflections [a]	37,083 (5,558)
Resolution (Å) [a]	43.35 – 2.13 (2.26 – 2.13)
R_{meas} (%) [a, b]	19.4 (166.4)
I/σ(I)*	8.69 (1.10)
$CC_{1/2}$ [a, c]	0.996 (0.416)
Completeness (%) [a]	97.3 (89.7)
Sigano [a]	1.405 (0.722)
Multiplicity [a]	6.97 (6.28)
Wilson B-factor (Å^2)	41.1
**Phasing statistics**	
No. of SeMet sites identified [d]	21
Overall Figure of Merit (FOM) [d]	0.392
FOM after density modification [d]	0.630
**Refinement**	
Resolution [a]	43.39 – 2.13 (2.18 – 2.13)
Unique reflections [a]	19,452 (2,544)
R_{work} (%) [a]	21.4 (29.6)
R_{free} (%) [a]	26.6 (34.9)
Number of atoms	3,000
B-factors (Å^2)	

overall	52.1
protein residues	52.3
ligands	53.1
water	43.4
Rmsd from ideal values [e]	
Bond length (Å)	0.005
Bond angles (º)	0.742
**Validation**	
Ramachandran plot [d]	
Favored (%)	97.4
Allowed (%)	2.3
Outliers (%)	0.3
Ramachandran plot *Z*-score, (Rmsd) [d]	
whole	-1.22
helix	-1.67
sheet	1.13
loop	-1.02
MolProbity Score [f, g]	1.55
MolProbity clashscore [f, g]	5.7

[a] data for the highest resolution shell in parenthesis
[b] $R_{meas}(I) = \sum_h [N/(N-1)]^{1/2} \sum_i | I_{ih} - \langle I_h \rangle | / \sum_h \sum_i I_{ih}$, in which $\langle I_h \rangle$ is the mean intensity of symmetry-equivalent reflections h, I_{ih} is the intensity of a particular observation of h and N is the number of redundant observations of reflection h (Diederichs and Karplus, 1997)
[c] $CC_{1/2} = (\langle I^2 \rangle - \langle I \rangle^2) / (\langle I^2 \rangle - \langle I \rangle^2) + \sigma^2_\varepsilon$, in which σ^2_ε is the mean error within a half-dataset (Karplus and Diederichs, 2012)
[d] calculated with PHENIX (Adams et al., 2010)
[e] Rmsd – root mean square deviation
[f] calculated with MOLPROBITY (Williams et al., 2018)
[g] Clashscore is the number of serious steric overlaps (> 0.4) per 1,000 atoms (Williams et al., 2018)

Supplemental Table S2. Statistical analysis of experimental data presented in Figure 2A.

Results of the One-way ANOVA comparing the average melting temperatures of HsPARP1 catalytic domain at the seven different 6(5H)-phenanthridinone concentrations:

source	sum of squares SS	degrees of freedom ν	mean square MS	F statistic	p-value
treatment	90.7125	6	15.1188	19.5899	8.1929e-06
error	10.0329	13	0.7718		
total	100.7455	19			

The highest 6(5H)-phenanthridinone concentration was only included in two experiments.

Results of the Tukey HSD test comparing all average melting temperatures of individual 6(5H)-phenanthridinone concentrations to the lowest concentration:

comparison against Tm[0.002 μM]	Tukey HSD Q statistic	Tukey HSD p-value
Tm[0.02 μM]	1.9333	0.7872806
Tm[0.2 μM]	0.0055	0.8999947
Tm[2 μM]	2.3987	0.6169576
Tm[20 μM]	9.0091	0.0010053
Tm[200 μM]	7.5906	0.0018610
Tm[2000 μM]	7.4211	0.0022705

Results of the One-way ANOVA comparing the average melting temperatures of the Ta-sro1 PARP domain at the seven different 6(5H)-phenanthridinone concentrations:

source	sum of squares SS	degrees of freedom ν	mean square MS	F statistic	p-value
treatment	0.6682	6	0.1114	1.4348	0.2697
error	1.0867	14	0.0776		
total	1.7549	20			

Supplemental Table S3. Statistical analysis of experimental data presented in Figure 2D.

Results of the One-way ANOVA comparing the average PARP activity of the different proteins:

source	sum of squares SS	degrees of freedom ν	mean square MS	F statistic	p-value
treatment	14.4572	7	2.0653	110.4077	2.4304e-12
error	0.2993	16	0.0187		
total	14.7565	23			

Results of the Tukey HSD test comparing the average PARP activities of all proteins against the BSA control:

comparison PARP activity BSA against	Tukey HSD Q statistic	Tukey HSD p-value
HsPARP1	21.7494	0.0010053
HsPARP1 1 mM 3AB	0.7050	0.8999947
HsPARP1 PARP L713F	22.7249	0.0010053
Ta-sro1	1.3580	0.8999947
Ta-sro1 WWE-PARP	1.7299	0.8999947
Ta-sro1 PARP	1.0047	0.8999947
no protein control	4.1770	0.1252163

Supplemental Table S4. List of oligonucleotides used in this work.

oligo name	purpose	orientation	sequence
LW1747	cloning of Ta-sro1 WWE-PARP (2-434) into pOPIN-F	fw	AAGTTCTGTTTCAGGGCCCGGAAAGGAAGACTGGAATGGTAC
LW874	cloning of Ta-sro1 PARP (246-434) into pOPIN-F	fw	AAGTTCTGTTTCAGGGCCCGattggccaacctgttg
LW1270	Ta-sro1 PARP reverse primer for cloning into pOPIN-F	rv	ATGGTCTAGAAAGCTTCAattggtcatggaaggtgctttg
LW1273	cloning of Ta-sro1 and Ta-SRO1 into pENTR4	fw	tacaaaaaagcaggctccacATGGAAAGGAAGACTGGAATGGTAC
LW1274	cloning of Ta-sro1 and Ta-SRO1 into pENTR4	rv	gaaagctgggtctagatatctcgagttGGAGGTGCTGCTCCCTCC
LW1225	site-directed mutagenesis of AtPARP2 E614Q	fw	gggatgttgttgtaCaaccaatatatagtc
LW1226	site-directed mutagenesis of AtPARP2 E614Q	rv	gactatatattggttGtacaacaacatccc

Supplemental Methods. Experimental procedures for protein expression, purification, and crystallization as well as the activity and binding assays.

Plant growth conditions

N. benthamiana seeds were sown on Flora clay (92% raised-bog peat, 0.15% salinity pH 5-6), stratified for 3 days at 4°C, and then cultivated under long day (16 h light, 8 h dark) conditions at 22 °C and approximately 30% relative humidity in a greenhouse under supplementary light from Tungsten lamps at approximately 200 µmol/ s m².

Molecular cloning

To generate His$_6$-tagged expression constructs, the coding sequences of Ta-sro1 WWE-PARP (residues 2-434) and Ta-sro1 PARP (residues 246-434) were cloned into *Kpn*I/*Hind*III-linearized pOPIN-F (Berrow et al., 2007) via Gibson assembly. The pET24 plasmid for expression of full-length Ta-sro1 was a kind gift from Prof. Guangmin Xia (Liu et al., 2014). The expression constructs for the catalytic domains of HsPARP1 (Langelier et al., 2012) and HsPARP10 (Kleine et al., 2008) have been described. For transient expression in *Nicotiana benthamiana*, the coding sequences of Ta-sro1 and Ta-SRO1 were cloned into *Nco*I/*Xho*I-linearized pENTR4 via Gibson assembly (residues 1-578, no stop codon). The pENTR4 AtPARP2 plasmid has been described (Chen et al., 2018). The AtPARP2 point mutation E^{614}Q was introduced in pENTR4 by site-directed mutagenesis. pENTR4-GFP-C3 (w393-1) was a gift from Eric Campeau & Paul Kaufman (Addgene plasmid # 17397; http://n2t.net/addgene:17397; RRID:Addgene_17397). The pENTR4 plasmids were recombined in Gateway™ LR reactions (Thermo Fisher Scientific) with pGWB414 (Nakagawa et al., 2007) to create expression constructs with a C-terminal 3xHA tag. To create expression constructs with a C-terminal eGFP tag, the respective pENTR4 plasmids were recombined in Gateway™ LR reactions with pK7FWG2 (Karimi et al., 2002).

Protein expression and purification

The Ta-sro1-His$_6$ full-length protein was expressed from pET24a in *E. coli* BL21 cells. The Ta-sro1 His$_6$-WWE-PARP and His$_6$-PARP constructs were expressed from pOPIN-F in *E. coli* SHuffle cells. The HsPARP1 catalytic domain construct was expressed from pET28 in *E. coli* SoluBL21 cells (Langelier et al., 2012). The GST-HsPARP10 catalytic domain containing amino acids 818-1025 was produced in *E. coli* BL21 cells essentially as described before (Kleine et al., 2008), with induction of protein expression at an OD_{600} of 0.6 with 1 mM IPTG, followed by 16 h incubation at 18 °C. Bacterial cultures were spun down (6000 x *g* / 4 °C / 15 min), followed by resuspension in lysis buffer [20 mM Tris-HCl pH 8.0, 150 mM NaCl, 0.1 mM EDTA pH 8.0, 5 mM DTT and 1x protease inhibitor cocktail (Sigma)], supplemented with 1 mg/mL Lysozyme (final concentration) and incubated on ice for 30 min. Cell suspensions were sonicated on ice (Branson Digital Sonifier 250 Cell Disruptor, 5 min at 20%, 30 s on/off rate). The cell lysates were centrifuged at 45000 x *g* for 45 min at 4°C to remove cell debris. The supernatant was used for affinity purification and incubated with Glutathione Sepharose 4B (Cytiva). The beads were washed in wash buffer (100 mM Tris-HCl pH 8.0, 120 mM NaCl) followed by elution using glutathione (20 mM in wash buffer, prepared fresh) and dialysis overnight [20 mM Tris-HCl pH 8.0, 150 mM NaCl, 1 mM DTT, 10% (v/v) glycerol].

For Ta-sro1 expression constructs, two to eight 1 L cultures were grown in LB medium at a temperature of 37 °C to an OD_{600} of 1.0 – 1.2. The cultures were cooled to 18 °C before expression was induced by the addition of 0.5 mM IPTG for 16 h. Cells were pelleted by centrifugation (5000 x *g* / 4 °C / 12 min) and the pellets were resuspended in buffer A [50 mM Tris-HCl pH 8.0, 0.3 M NaCl, 20 mM imidazole, 5% (v/v) glycerol, 50 mM glycine] supplemented with 0.1% Polyethylenimine and 1x cOmplete™ EDTA-free protease inhibitor cocktail (Roche). Cells lysis was induced by addition of Lysozyme (1 mg/mL final concentration / 25 °C / 15 min) followed by sonication on ice (Branson 150D Sonifier, 2x 10 min, level 3-4). Insoluble proteins and cell debris were removed by centrifugation (30000 x *g* / 4 °C / 30 min) and the supernatant was loaded

onto a 5 mL HisTrap HP IMAC column (Cytiva). The column was washed with buffer A until the A_{280} reached 25 mAU and proteins were eluted using buffer B [50 mM Tris-HCl pH 8.0, 0.3 M NaCl, 0.5 M imidazole, 5% (v/v) glycerol, 50 mM glycine]. The elution from the IMAC column was injected onto a size exclusion chromatography column [Superdex 75 or Superdex 200 26/60 PG column (Cytiva) pre-equilibrated with 20 mM HEPES-NaOH pH 7.5, 150 mM NaCl]. Proteins eluting from the column were concentrated by ultrafiltration on Vivaspin 20 and 2 columns (Sartorius) with a 5 kDa molecular weight cut-off. The Selenomethionine-labeled Ta-sro1 PARP domain was produced using feedback inhibition (Van Duyne et al., 1993) and purified as described above. For crystallization, the His_6-tag was cleaved using 3C protease. The protein was run through a 5 mL HisTrap HP IMAC column in buffer A to remove the His_6-tag and residual un-cleaved fusion protein, followed by injection onto the Superdex 75 26/60 PG column and eluted and concentrated as above.

Protein crystallization and structure determination

Crystals of native and Selenomethionine-labeled Ta-sro1 PARP domain formed in 0.1 M MES-KOH pH 5.8, 0.2 M ammonium sulfate, 17% (w/v) PEG 3350 at a protein concentration of 23.3 mg/mL in a vapor diffusion setup at 291 K. The crystals were transferred to cryo-protectant solution [0.1 M MES-KOH pH 5.8, 0.2 M ammonium sulfate, 17% (w/v) PEG 3350, 25% (v/v) ethylene glycol] and flash-frozen in liquid nitrogen. Diffraction data was collected at beamline 14.2 of the BESSY II synchrotron, Berlin at 100 K. The data were processed with XDS (Kabsch, 2010; Sparta et al., 2016) and the PARP domain structure was solved by single anomalous diffraction phasing using the AutoSol wizard in PHENIX (Adams et al., 2010). The final model was obtained by iterative building and refinement cycles using Coot (Emsley et al., 2010) and PHENIX (Afonine et al., 2012). In final stages of refinement, TLS refinement (Winn et al., 2001) was performed as implemented in PHENIX. The model was validated using Molprobity (Chen et al., 2010) and Coot (Emsley et al., 2010). The statistics for X-ray data collection and the refined model are given in Supplemental Table S1. Reflection data and the Ta-sro1 PARP domain structure have been deposited at the Protein Data Bank with identifier 7PLQ. Diffraction images have been deposited at www.proteindiffraction.org under doi:10.18430/M37PLQ. 3D visualizations of protein structures were prepared using PyMol software v1.7.2 (https://sourceforge.net/projects/pymol/).

Thermal stability assay

Thermal stability assays were performed in a MX3005 qPCR System (Agilent Technologies) with thermal denaturation ranging from 25 °C to 95 °C in 0.5 °C steps. All samples were measured as technical triplicates and contained 25 mM NaCl, 75 mM Tris-HCl pH 7.5 and 6.6x SYPRO™ Orange (Thermo Fisher Scientific) in a final volume of 20 µL. Negative controls additionally contained the highest concentration of 6(5H)-phenanthridinone (Sigma-Aldrich) but no protein or only protein without ligand. All other samples contained the same amount of protein (0.11 mg/mL) and seven 6(5H)-phenanthridinone concentrations ranging from 2 nM to 2 mM. The global minimum of the negative derivative of fluorescence over temperature was considered the melting temperature. Melting temperatures were averaged from three technical replicates and three independent experiments and were plotted against ligand concentrations.

NAD^+ binding and auto-ADP-ribosylation assays

NAD^+ binding was tested by spotting the proteins on nitrocellulose membrane followed by incubation with radiolabelled NAD^+. Serial dilutions of the proteins were spotted, with 500 ng protein as highest concentration followed by 1:2 dilutions. After spotting the proteins, the membrane was allowed to dry before blocking in 5% non-fat milk in PBST. The blocked membrane was washed extensively in PBST before addition of 10 µCi [^{32}P]-β-NAD^+ (Hartmann Analytic) in PBST. After one h incubation at RT, the membrane was washed again in PBST, followed by exposure to X-ray film of the dried membrane. Protein auto-ADP-ribosylation assays were performed at 37 °C for 30 min. Reactions were carried out in 30 µL volume containing 50 mM Tris-

HCl pH 8.0, 0.2 mM DTT, 4 mM $MgCl_2$ and 50 µM β-NAD^+ (Sigma-Aldrich) and 1 µCi [^{32}P]-β-NAD^+ (Hartmann Analytic). Reactions were stopped by adding SDS sample buffer, heated for 5 min at 95 °C and analyzed using SDS-PAGE. Gels were dried and incorporated radioactivity was analyzed by exposure of the dried gel to X-ray film.

PARP activity assay
The PARP activity assay was performed with the PARP Universal Colorimetric Assay Kit (Trevigen) according to the manufacturer's manual, section 'PARP Inhibitor Assay Protocol'. 0.5 µg of protein was added per well and each reaction was performed in triplicate. Within the recommended assay time of 1 h, the two HsPARP1 reactions produced A_{450} values that were too high to be measured by the Tecan Infinite F50 plate reader. Therefore, the reactions were already stopped and quantified after 15 min.

Agrobacterium-mediated transient expression
Binary vectors were transformed into *Agrobacterium tumefaciens* strain GV3101 pMP90. *A. tumefaciens* strains were grown on selective LB plates, resuspended in 10 mM $MgCl_2$ 10 mM MES-KOH pH 5.6 and incubated with 100 µM acetosyringone for 2 h at RT. Prior to infiltration, each strain was mixed with *A. tumefaciens* strain GV3101 pMP90 expressing the silencing suppressor 19K at a ratio of 1:1.5[19K]. The cultures were infiltrated into leaves of 4-5 week-old *N. benthamiana* plants using a needleless syringe and leaf material for protein extraction or confocal microscopy was harvested 48–72 h later.

Confocal microscopy
Leaf discs excised from *N. benthamiana* were mounted on microscope slides in 50% (v/v) glycerol and the subcellular localization of GFP-tagged proteins was analyzed using a Zeiss LSM 780 confocal microscope. Excitation wavelength and emission collection windows were 488/493-545 nm for GFP and 561/687-730 nm for chlorophyll. Images were acquired and analyzed using Zeiss Zen software.

Protein extraction and immunopurification from leaf tissue
Protein extracts were prepared by grinding *N. benthamiana* leaf material in liquid nitrogen to a fine powder followed by resuspension in extraction buffer [50 mM Tris-HCl pH7.4, 150 mM NaCl, 10% (v/v) glycerol, 1 mM EDTA, 5 mM DTT, 1× protease inhibitor cocktail (Sigma-Aldrich #P9599), 0.2% NP-40] at a ratio of 2 mL buffer per 1 g leaf material. Crude protein extracts were centrifuged at 20000 x *g* / 4 °C / 20 min and the supernatant was split into two samples for immunoprecipitation. One sample was supplemented with 300 µM β-NAD^+ (Sigma-Aldrich) to enhance auto-ADP-ribosylation. For immunoprecipitation, 15 µL of α-GFP-nanobody:Halo:His_6 magnetic beads (Chen et al., 2018) were added to 1.4 mL of the supernatant. The samples were incubated on a rotating wheel at 4 °C for 2 h followed by collection of the beads using a magnetic sample tube rack. The beads were washed 3 times with 1 mL extraction buffer and then boiled in 40 µL SDS sample buffer to elute protein from the beads.

SDS-PAGE and immunoblotting
Protein samples were separated by SDS-PAGE and stained using Coomassie Brilliant Blue. For immunoblots, proteins were electro-blotted onto PVDF membrane and blocked with a solution of 5% non-fat dry milk in TBST for 1 h at RT. The membranes were incubated with α-GFP antibody (Amsbio, TP401, 1:5000 dilution) or α-pan-ADP-ribose binding reagent (Sigma-Aldrich, MABE1016, 1:4000 dilution) in 2.5% non-fast dry milk over night at 4 °C. Proteins were detected using a HRP-coupled goat-anti-rabbit secondary antibody and X-ray films.

Sequence analysis and alignments
Disordered regions of Ta-sro1 were predicted using the PONDR VL3-BA algorithm (Xue et al., 2010). SRO proteins were identified using JACKHMMER (Potter et al., 2018) with the Ta-sro1 PARP domain as a query and the following settings: HmmerWeb version 2.41.2, database Reference proteomes, restricted to *Viridiplantae* (taxid:33090), E-values Sequence = 0.0001, Hit = 0.0003. The sequence conservation logo was generated with Skylign (Skylign.org by Jody Clements, Travis Wheeler & Robert Finn, Interactive logos for alignments and profile HMMs, http://skylign.org/, CC BY 3.0 license).

Accession Numbers
Sequence data from this article can be found in the EMBL/GenBank data libraries under accession number(s) AEK94072.1 (Ta-sro1), AT1G32230 (RCD1), AT1G23550 (SRO2). Reflection data and the Ta-sro1 PARP domain structure have been deposited at the Protein Data Bank with identifier 7PLQ. Diffraction images have been deposited at www.proteindiffraction.org under doi:10.18430/M37PLQ.

Supplemental References

Afonine PV, Grosse-Kunstleve RW, Echols N, Headd JJ, Moriarty NW, Mustyakimov M, Terwilliger TC, Urzhumtsev A, Zwart PH, Adams PD (2012) Towards automated crystallographic structure refinement with phenix.refine. Acta Crystallogr D Biol Crystallogr **68**: 352–367.

Berrow NS, Alderton D, Sainsbury S, Nettleship J, Assenberg R, Rahman N, Stuart DI, Owens RJ (2007) A versatile ligation-independent cloning method suitable for high-throughput expression screening applications. Nucleic Acids Res **35**: e45.

Chen VB, Arendall WB, Headd JJ, Keedy DA, Immormino RM, Kapral GJ, Murray LW, Richardson JS, Richardson DC (2010) MolProbity: all-atom structure validation for macromolecular crystallography. Acta Crystallogr D Biol Crystallogr **66**: 12–21.

Diederichs K., Karplus PA (1997) Improved R-factors for diffraction data analysis in macromolecular crystallography. Nat Struct Biol **4**: 269–275.

Emsley P, Lohkamp B, Scott WG, Cowtan K (2010) Features and development of Coot. Acta Crystallogr D Biol Crystallogr **66**: 486–501.

Kabsch W (2010) XDS. Acta Crystallogr D Biol Crystallogr **66**: 125–132.

Karimi M, Inzé D, Depicker A (2002) GATEWAY vectors for Agrobacterium-mediated plant transformation. Trends Plant Sci **7**: 193–195.

Karplus PA, Diederichs K (2012) Linking crystallographic model and data quality. Science **336**: 1030–1033.

Langelier MF, Planck JL, Roy S, Pascal JM (2012) Structural basis for DNA damage-dependent poly(ADP-ribosyl)ation by human PARP-1. Science **336**: 728–732.

Nakagawa T, Suzuki T, Murata S, Nakamura S, Hino T, Maeo K, Tabata R, Kawai T, Tanaka K, Niwa Y, Watanabe Y, Nakamura K, Kimura T, Ishiguro S (2007) Improved Gateway binary vectors: high-performance vectors for creation of fusion constructs in transgenic analysis of plants. Biosci Biotechnol Biochem **71**: 2095–2100.

Sparta KM, Krug M, Heinemann U, Mueller U, Weiss MS (2016) XDSAPP2.0. J Appl Crystallogr **49**: 1085–1092.

Van Duyne GD, Standaert R, Karplus PA, Schreiber SL, Clardy J (1993) Atomic structures of the human immunophilin FKBP-12 complexes with FK506 and rapamycin. J Mol Biol **229**: 105–124.

Williams CJ, Headd JJ, Moriarty NW, Prisant MG, Videau LL, Deis LN, Verma V, Keedy DA, Hintze BJ, Chen VB, Jain S, Lewis SM, Arendall WB, Snoeyink J, Adams PD, Lovell SC, Richardson JS, Richardson DC (2018) MolProbity: More and better reference data for improved all-atom structure validation. Protein Sci **27**: 293–315.

Winn MD, Isupov MN, Murshudov GN (2001) Use of TLS parameters to model anisotropic displacements in macromolecular refinement. Acta Crystallogr D Biol Crystallogr **57**: 122–133.
